# Hypothalamic volume is associated with body mass index

**DOI:** 10.1016/j.nicl.2023.103478

**Published:** 2023-07-24

**Authors:** Stephanie S.G. Brown, Margaret L. Westwater, Jakob Seidlitz, Hisham Ziauddeen, Paul C. Fletcher

**Affiliations:** a Department of Psychiatry, University of Cambridge, Addenbrookes Hospital, Cambridge CB2 0QQ, United Kingdom; b Department of Psychiatry, University of Oxford, Warneford Hospital, Oxford OX3 7JX, United Kingdom; c Department of Child and Adolescent Psychiatry and Behavioral Science, Children’s Hospital of Philadelphia, Philadelphia, PA, USA; d Department of Psychiatry, University of Pennsylvania, Philadelphia, PA, USA; e Lifespan Brain Institute of Children’s Hospital of Philadelphia and University of Pennsylvania, Philadelphia, PA, USA; f Wellcome Trust MRC Institute of Metabolic Science, University of Cambridge, Cambridge Biomedical Campus, Cambridge CB2 0QQ, United Kingdom; g Cambridgeshire and Peterborough NHS Trust, United Kingdom

**Keywords:** Hypothalamus, Body Mass Index (BMI), Structural MRI, Obesity, Overweight, Anorexia nervosa, Inflammation

## Abstract

The hypothalamus is an important neuroendocrine hub for the control of appetite and satiety. In animal studies it has been established that hypothalamic lesioning or stimulation causes alteration to feeding behaviour and consequently body mass, and exposure to high calorie diets induces hypothalamic inflammation. These findings suggest that alterations in hypothalamic structure and function are both a cause and a consequence of changes to food intake. However, there is limited *in vivo* human data relating the hypothalamus to obesity or eating disorders, in part due to technical problems relating to its small size. Here, we used a novel automated segmentation algorithm to exploratorily investigate the relationship between hypothalamic volume, normalised to intracranial volume, and body mass index (BMI). The analysis was applied across four independent datasets comprising of young adults (total n = 1,351 participants) spanning a range of BMIs (13.3 – 47.8 kg/m^2^). We compared underweight (including individuals with anorexia nervosa), healthy weight, overweight and obese individuals in a series of complementary analyses.

We report that overall hypothalamic volume is significantly larger in overweight and obese groups of young adults. This was also observed for a number of hypothalamic sub-regions. In the largest dataset (the HCP-Young Adult dataset (n = 1111)) there was a significant relationship between hypothalamic volume and BMI.

We suggest that our findings of a positive relationship between hypothalamic volume and BMI is potentially consistent with hypothalamic inflammation as seen in animal models in response to high fat diet, although more research is needed to establish a causal relationship. Overall, we present novel, *in vivo* findings that link elevated BMI to altered hypothalamic structure. This has important implications for study of the neural mechanisms of obesity in humans.

## Highlights

• The hypothalamus is a key brain region for the control of appetite.
• Using structural MRI, the hypothalamus was segmented in a total of 1,351 participants of varying body mass indices.
• We show that the volume of the hypothalamus is significantly increased in people who are overweight or obese.

## 1 Introduction

The hypothalamus and associated neuronal circuitry is crucial to maintaining energy balance through appetite regulation (Sohn, 2015, King, 2006). Its role in eating behaviour is long-established, with initial experiments observing the profound effects of lesions or electrical stimulation on feeding behaviour (Williams et al., 2001). In recent decades, investigations into the hypothalamus have revealed key neuronal populations within the hypothalamus which regulate appetite, and further, the regulatory effects of peripherally produced cytokines and hormones such as leptin and insulin on hypothalamic function (Coll et al., 2004).

### 1.1 Hypothalamic circuitry and neuronal populations

The hypothalamus is located ventrally to the thalamus and forms part of the diencephalon. It connects extensively to cortical, subcortical and brainstem regions (2000). There is abundant evidence that neuronal populations within the hypothalamus, located within the arcuate nucleus and the paraventricular nucleus (PVN), make major contributions to appetite regulation. The arcuate nucleus has been particularly well-characterised in its relation to appetite, with its two opposite-acting distinct neuronal populations: the orexigenic neuropeptide Y/agouti-related peptide (AgRP) neurons and the anorexigenic pro-opiomelancortin (POMC) neurons. The appetite-suppressing function of POMC neurons was demonstrated in 1999 (Yaswen et al., 1999), with POMC knockout resulting in obesity and hyperphagia in mouse models. Moreover, stimulation of POMC neurons leads to reduction in food intake (Zhan et al., 2013). The AgRP orexigenic portion of the arcuate nucleus is similarly critical for food-intake regulation, as confirmed by ablation studies (Wu et al., 2009, Wu et al., 2012) and by observations that AgRP stimulation results in marked increase in feeding behaviour (Aponte et al., 2011). This AgRP-mediated stimulation of food intake is thought to be predominantly controlled via GABAergic neurotransmission (Tong et al., 2008), which is propagated to the POMC population, forming circuitry within the arcuate nucleus which regulates increased appetite through inhibition of energy expenditure (Cowley et al., 2001). AgRP/NPY neurons also antagonise MC4R receptors to increase appetite via inverse agonism (Essner et al., 2017). The PVN, similarly to POMC neuronal function, is thought to also underlie anorexigenic signalling within the hypothalamus. In clinical syndromes such as Prader-Willi and Sim 1 haploinsufficiency, where genetic development of the PVN is specifically detrimentally impacted, hyperphagia and genetically-determined obesity are characteristic (Michaud, 2001, Brown et al., 2022, Hinton et al., 2006, Holland, 1993, Holsen et al., 2006). Orexin A and B (also known as hypocretin 1 and 2) are expressed in the lateral region of the hypothalamus, and these peptides also control feeding behaviour. Orexin-producing neurones are solely hypothalamic and form extensive and reciprocal connections to the solitary nucleus within the medulla, which receives vagal afferent innervation from the gut (Waise et al., 2018). It is via this mechanism, and connectivity to glycaemic-sensing neurones (Koekkoek et al., 2017), that orexin-producing neurones receive inhibitory input after food ingestion to signal satiety. The lateral hypothalamus, paraventricular and arcuate nuclei therefore comprise the predominant food-related homeostatic control centres of the hypothalamus, with disruption to any area of this internal or externally connected circuitry resulting in marked changes to appetite and body mass.

### 1.2 Body mass index and mental health

Complex relationships exist between eating behaviour, weight, psychiatric illness and physical pathophysiology. Current estimations suggest that over 1.9 billion people worldwide are either overweight or obese, with over 4 million deaths per year being attributable to being overweight according to the World Health Organisation, 2021. With higher body mass index (BMI) comes a significantly increased risk for cardiovascular disease, encompassing stroke, heart disease, and type II diabetes, with all of its associated risks. Being overweight also causes musculoskeletal complications and is linked to prevalence of some cancers, such as breast, liver and colon (Bajaj et al., 2018, Freisling et al., 2017). Evidence generally suggests that greater excess weight translates into greater risk for the development of these pathologies. Taken together, the health impact of climbing levels of obesity worldwide is striking. There is also a bidirectional link between higher BMI and mental illness, compounding negative health outcomes (Avila et al., 2015). Changes in one’s BMI, including significant weight gain and weight loss, have been associated with various mental health conditions, most notably eating disorders and depression (Yilmaz et al., 2019, Pereira-Miranda et al., 2017) which profoundly reduce one’s quality of life (Avila et al., 2015).

The eating disorders anorexia nervosa (AN) and bulimia nervosa (BN) affect individuals across the BMI-spectrum, and they share numerous symptoms, including a fear of weight gain and restricted eating. While the presence of underweight is necessary for a diagnosis of AN, BN patients have a wide range of BMIs (>18.5 kg/m^2^), and increased weight suppression, or the difference between one’s current weight and their highest past weight at adult height, represents a common risk factor for both conditions (Stice et al., 2020). AN and BN have high rates of comorbidity with various medical (e.g., gastrointestinal problems, hypokalaemia, osteopenia) and psychiatric problems (suicidality), which contributes to increased mortality among affected individuals (Schmidt et al., 2016). Growing evidence implicates metabolic perturbations in the pathogenesis of these illnesses (Watson et al., 2019), and altered hypothalamic functioning has been observed in animal models (Sutton Hickey, 2022, Adan, 2011) and neuroimaging studies of gustatory learning and metabolic processing in AN (Frank et al., 2018, Simon et al., 2020). Intriguingly, patients with both AN and obesity show blunted hypothalamic responses to intra-gastric glucose infusion, which corresponded to reduced nucleus accumbens and amygdala activity in AN (Simon et al., 2020). These data further support appetite and its regulation as a crucial area of study if we are to understand the underlying mechanisms of health-harming weight changes.

### 1.3 Imaging the hypothalamus

Much of the existing body of evidence for the role of the hypothalamus in appetite regulation originates from animal studies (Sohn, 2015). This is due to difficulties in imaging and studying the hypothalamus in humans, including a lack of defined contrast in structural images in this area of the brain, meaning that the hypothalamus and its constituent functional nuclei are challenging to delineate. Some studies have, however, manually segmented this region in human MRI scans *in vivo* (Baroncini et al., 2012, Bocchetta et al., 2015, Makris et al., 2013). Using this approach, researchers have found qualitatively reduced hypothalamic volumes in a small sample of frontotemporal dementia patients with severe eating disturbance (Bocchetta et al., 2015). A limitation of this approach however is that manual tracing of the hypothalamus is time-consuming and can be subject to inter-rater unreliability, and therefore has reduced suitability for the large neuroimaging datasets that are required for adequate statistical power and result reproducibility. Examining volumetrics of specialised brain regions using MRI is a useful, albeit non-specific tool in understanding healthy and pathological neural mechanisms. In addition to clear atrophic disease processes, regional structural MR volumetrics are known to indicate volume of cellular nuclei, local cell number and spatial clustering characteristics of cells as measured by two-photon microscopy (Asan et al., 2021). Therefore, in an anatomical region such as the hypothalamus, with highly localised neuroendocrine function and specialised cellular populations, size may reflect such functionally relevant domains. A recently developed algorithm, created using convolutional neural networks, now allows for automated volumetric segmentation of the hypothalamus with high reported accuracy (Billot, 2020), which presents the opportunity for larger-scale investigation into the hypothalamic structure in humans *in vivo*. In the current study we sought to exploit this by applying it to previously acquired structural MRI observations using a set of independent datasets from individuals across a range of BMI scores. The first (Westwater et al., 2021) compared underweight individuals suffering from anorexia nervosa with normal weight healthy controls as well as normal weight people with bulimia nervosa. The second used data from a study of overweight and obese individuals (Westwater, 2019) using normative data (Kiddle et al., 20182018) as a control. The third analysis explored an openly available dataset (Strike et al., 20192019) and evaluated hypothalamic volume across a range of BMI categories: underweight, normal weight, overweight and obese.

We hypothesised in this exploratory study that the hypothalamus may be structurally related to body mass index. Given the roles of the arcuate nucleus in POMC signalling (segmented within the tubular inferior subunit), the lateral hypothalamus in orexin signalling (segmented within the tubular superior and posterior subunits) and the paraventricular nucleus in anorexigenic signalling (segmented within the tubular superior subunit), we specifically predicted that these regions, encompassed by the aforementioned algorithm-segmented nuclei, may be preferentially affected in people with a higher deviation from normal body mass. Previous human neuroimaging investigation of the hypothalamus in obesity has demonstrated that hypothalamic resting-state functional connectivity is enhanced with feeding-related areas and diminished with regions of cognitive food intake control in those with higher BMI (Le et al., 2020). Greater BMI has also been reported to be associated with alterations to the hypothalamic microstructure (Thomas et al., 2019). The existing evidence-base therefore supported a hypothesis of potential volumetric alterations to the hypothalamus in tandem with higher or lower than normal BMI.

## 2 Methods and materials

### 2.1 Participants

Data for this study were drawn from four existing MRI datasets comprised of young adult participants: Overweight participants with a BMI 25 – 29.9 kg/m^2^ (n = 55) and obese participants with a BMI over 30 kg/m^2^ (n = 35) (Westwater, 2019, Medic et al., 2016), normal weight control participants matched to the obese/overweight group from the NeuroScience in Psychiatry Network (NSPN) U-Change project (n = 66) (Kiddle et al., 20182018), women with anorexia nervosa (AN) (n = 21), bulimia nervosa (BN) (n = 33) and age-matched controls (n = 30) (Westwater et al., 2021), and the Human Connectome Project (HCP) Young Adult dataset (n = 1111) (Strike et al., 20192019). BMI was calculated using measured body weight and height for all participants in all datasets.

### 2.2 MRI acquisition

Participants within the smaller obesity, NSPN and AN/BN datasets underwent MRI scanning at the Wolfson Brain Imaging Centre in Cambridge, UK. T1-weighted structural images were acquired on a 3 T Siemens MAGNETOM Skyra system, which was fitted with a 32-channel, GRAPPA (generalized autocalibrating partial parallel acquisition) parallel-imaging head coil, using the following parameters: TE = 2.95 ms; TR = 2300 ms; flip angle = 9°; acquisition matrix = 256 × 256 mm, 1.0 mm isotropic resolution.

Publicly available data from the human connectome project (HCP; https://www.humanconnectome.org/), comprised MRI data from 1,111 individuals (605 female) from 457 unique families (including 170 dizygotic twins, 286 monozygotic twins, 576 non-twin siblings, and 25 non-sibling familial relations) with mean age 28.8 years (SD = 3.7, range = 22–37). As previously described in detail (Van Essen et al., 2013, Glasser et al., 2013), T1-weighted and T2-weighted structural images were.

acquired on a 3 T Siemens Skyra employing a 32-channel head coil.

### 2.3 MRI data analysis

**Fig. 1:**
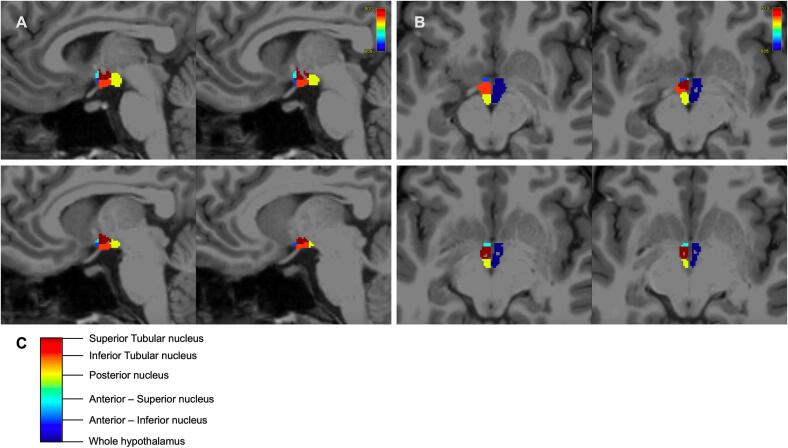
**A** Sagittal brain slices showing segmented hypothalamic nuclei **B** Axial brain slices showing segmented hypothalamic nuclei and whole hypothalamus.

T_1_-weighted images were pre-processed using the FreeSurfer v7.0 ‘recon-all’ pipeline (https://surfer.nmr.mgh.harvard.edu/fswiki/recon-all). The pre-processed structural data were then segmented to extract the whole hypothalamus and hypothalamic nuclei using an automated tool, which is based on a deep convolutional neural network in FreeSurfer development version 7 (Billot, 2020) (Fig. 1). Per hemisphere, subnuclei per hemisphere were segmented and labelled as the following: anterior-inferior, anterior–superior, posterior, tubular inferior and tubular superior. These segmented subregions overlap with more granular nuclei of the hypothalamus, as reported in Billot *et al,* 2020 (Billot, 2020), but do not segment functional or anatomical regions in an isolated manner. Hypothalamic volume data was also normalised to intracranial volume (ICV) using proportional correction (Voevodskaya, 2014). Acquisitions segmentations were manually spot-inspected for quality assurance. Visual inspection was carried out by using overlay onto the T_1_-weighted images and inspecting good quality alignment to neuroanatomy.

### 2.4 Statistical analysis

Between group analyses were carried out using non-parametric t-testing (Mann-Whitney U) and corrected for multiple comparisons using false discovery rate (FDR). Association analyses were carried out using linear regression, to assess potential relationships between the whole hypothalamic volume and BMI. The five subunit regions of the hypothalamus and the whole hypothalamus were compared between groups bilaterally, to take into account possible hemisphere-specific effects.

### 2.5 Data availability statement

The Human Connectome Project (HCP) Young Adult dataset is a publicly available resource that may be accessed at: https://www.humanconnectome.org/study/hcp-young-adult. Raw structural images from the NSPN cohort may be downloaded from: https://nspn.org.uk. Data from the Cambridge obesity and eating disorder studies is available upon reasonable request to researchers who have ethical approval for research on these topics.

### 2.6 Ethics statement

Fully informed written consent was obtained from all participating individuals, and the protocols were approved by the following regional ethics committees: Cambridge University Psychology Research Ethics Committee and Cambridge East Research Ethics Committee.

## 3 Results

### 3.1 Participant demographics

Participant demographic information is summarised for the smaller datasets in Table 1 and for the replication HCP Young Adult dataset in Table 3. In the AN, BN and matched control datasets, all participants were female. In the overweight group, 17 participants were male and 38 were female; in the obese group, 23 participants were male and 12 were female; of the matched controls, 23 were male and 43 were female. All participants were in the mean age range of 20–30 years old. Age was higher in the obese group compared to their control group by a mean of 3.3 years. In the eating disorder sample, all groups were matched on age, but BMI was significantly different in all groups except for BN compared to controls. In the HCP Young Adult dataset, age was not significantly different between BMI groupings of underweight (BMI < 18.49 kg/m^2^), normal weight (BMI 18.5 – 24.9 kg/m^2^), overweight (BMI 25 – 29.9 kg/m^2^) and obese (BMI 30 + kg/m^2^). Of the underweight group, 2 were male and 17 were female; of the normal weight group, 186 were male and 288 were female; of the overweight group, 215 were male and 160 were female; of the obese group, 103 were male and 140 were female.

**Table 1:** Cambridge cohorts participant demographic information.

	Control mean (SD)	Overweight mean (SD)	Obese mean (SD)	BN mean (SD)	AN mean (SD)	AN/BN matched control mean	Overweight-Control p-value	Obese-Control p-value	BN-Control p-value	AN-Control p-value
Age (Years)	24.2 (5.0)	25.6 (5.3)	27.5 (6.1)	23.2 (3.8)	24.7 (4.8)	23.9 (3.5)	–	**	–	–
Body Mass Index	22.5 (1.6)	26.9 (1.6)	33.6 (3.9)	21.8 (2.4)	16.2 (1.3)	21.9 (2.1)	***	***	–	***

Means, standard deviations (SD) and *t*-test p-value results of participant demographics for overweight, obese, bulimia nervosa (BN), anorexia nervosa (AN) and matched control groups ((- = p > 0.5, * = p < 0.05, ** = p < 0.01, *** = p < 0.001). Control groups = BMI 18.5 – 24.9, overweight = BMI 25 – 29.9, obese = BMI 30 +.

### 3.2 Hypothalamic volumetrics

#### 3.2.1 Primary Cambridge cohorts

**Fig. 2:**
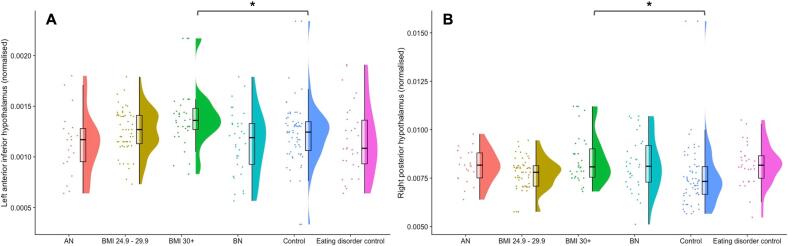
Anorexia nervosa (AN), bulimia nervosa (BN), eating disorder matched control, overweight, obese and overweight/obese control groups (Control groups = BMI 18.5 – 24.9, overweight = BMI 25 – 29.9, obese = BMI 30 +.): **A** left anterior-inferior and **B** right posterior hypothalamic substructures normalised to intracranial volume (ICV). Left anterior-inferior and right posterior hypothalamic nuclei were significantly larger in those with body mass index (BMI) >30 compared to healthy weight controls. (* = p^corr^ < 0.05).

Non-parametric t-testing and correction for multiple comparison using false discovery rate (FDR) revealed that the volume of the left anterior-inferior and right posterior hypothalamic substructures normalised to ICV were significantly larger in the obesity group compared to their control group (p^corr^ < 0.05). There were no significant differences between the matched control group and the AN or BN groups in hypothalamic volume. The AN group exhibited smaller left tubular superior nuclei, right anterior superior nuclei and whole right hypothalami than their matched control group when volume was not normalised to ICV or corrected for multiple comparisons, ICV and FDR correction removed this effect. Anterior-inferior and posterior hypothalamic volumes are shown in Fig. 2 and full statistical results are reported in Table 2. Whole hypothalamic data for the combined Cambridge cohorts were regressed against BMI, showing a trend towards a significant relationship in the right whole hypothalamus (p = 0.06, r^2^ = 0.015) and no relationship between BMI and volume in the left hypothalamus (p = 0.29, r^2^ = 0.005). Statistical results for the obese, AN, BN and control groups without correction for ICV or FDR are reported in Supplementary Table 1.

**Table 2:** Cambridge cohorts: Hypothalamic nuclei volumetric between group results normalised to ICV.

	Control mean (SD)	Overweight mean (SD)	Obese (SD)	BN mean (SD)	AN mean (SD)	AN/BN matched control mean (SD)	Overweight-Control p-value (FDR)	Obese-Control p-value (FDR)	BN-Control p-value (FDR)	AN-Control p-value (FDR)
Hypothalamic volumes (left hemisphere)										
Anterior-inferior	0.0012 (0.0003)	0.0013 (0.0002)	0.0014 (0.0003)	0.0011 (0.0003)	0.0011 (0.0003)	0.0012 (0.0003)	–	* (*)	–	–
Anterior-superior	0.0017 (0.0004)	0.0017 (0.0002)	0.0017 (0.0003)	0.0015 (0.0002)	0.0014 (0.0003)	0.0015 (0.0003)	–	–	–	–
Posterior	0.0081 (0.0019)	0.0079 (0.0007)	0.0083 (0.0009)	0.0083 (0.0012)	0.0080 (0.0009)	0.0081 (0.0010)	–	–	–	–
Tubular inferior	0.0092 (0.0024)	0.0092 (0.0010)	0.0098 (0.0009)	0.0100 (0.0010)	0.0100 (0.0010)	0.0096 (0.0009)	–	–	–	–
Tubular superior	0.0077 (0.0018)	0.0077 (0.0007)	0.0077 (0.0008)	0.0077 (0.0010)	0.0074 (0.0007)	0.0078 (0.0008)	–	–	–	–
Whole	0.0280 (0.0065)	0.0276 (0.0019)	0.0289 (0.0016)	0.0287 (0.0024)	0.0280 (0.0022)	0.0282 (0.0024)	–	–	–	–
Hypothalamic volumes (right hemisphere)										
Anterior-inferior	0.0011 (0.0003)	0.0011 (0.0002)	0.0011 (0.0002)	0.0009 (0.0002)	0.0010 (0.0002)	0.0009 (0.0002)	–	–	–	–
Anterior-superior	0.0017 (0.0005)	0.0017 (0.0002)	0.0017 (0.0003)	0.0016 (0.0003)	0.0015 (0.0003)	0.0016 (0.0003)	–	–	–	–
Posterior	0.0076 (0.0018)	0.0077 (0.0008)	0.0085 (0.0013)	0.0082 (0.0014)	0.0081 (0.0009)	0.0082 (0.0011)	–	** (*)	–	–
Tubular inferior	0.0086 (0.0019)	0.0087 (0.0008)	0.0089 (0.0010)	0.0089 (0.0009)	0.0088 (0.0009)	0.0088 (0.0008)	–	–	–	–
Tubular superior	0.0083 (0.0023)	0.0082 (0.0008)	0.0082 (0.0008)	0.0081 (0.0010)	0.0078 (0.0009)	0.0080 (0.0008)	–	–	–	–
Whole	0.0275 (0.0065)	0.0274 (0.0021)	0.0285 (0.0019)	0.0278 (0.0028)	0.0272 (0.0023)	0.0275 (0.0023)	–	–	–	–

Means, standard deviations (SD) and *t*-test p-value results of hypothalamic volumes as normalised^§^ to intracranial volume (ICV) [^§^Hypothalamic volume as a percentage of ICV]; (- = p > 0.5, * = p < 0.05, ** = p < 0.01, *** = p < 0.001). Bracketed significance indicator denotes FDR corrected p-value. Control groups = BMI 18.5 – 24.9, overweight = BMI 25 – 29.9, obese = BMI 30 +.

**Table 3:** HCP Young Adult participant demographic information.

	Underweight mean (SD)	Normal weight mean (SD)	Overweight mean (SD)	Obese mean (SD)
Age (years)	29.4 (3.6)	28.5 (3.7)	28.8 (3.6)	29.0 (3.6)
Body Mass Index	17.7 (0.7)	22.4 (1.7)	27.1 (1.4)	34.4 (1.4)

Means and standard deviations (SD) of participant demographics for HCP young adults grouped by BMI. Underweight = BMI < 18.49, normal weight = BMI 18.5 – 24.9, overweight = BMI 25 – 29.9, obese = BMI 30 +.

#### 3.2.2 HCP replication cohort

**Fig. 3:**
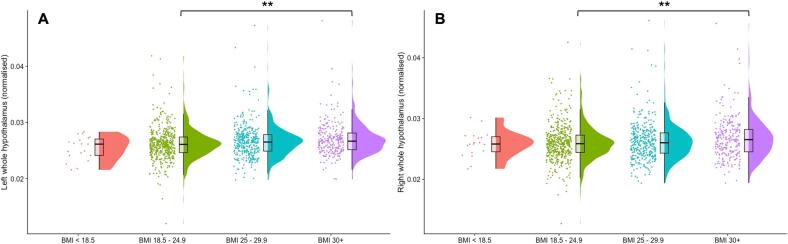
Whole hypothalamic volumes (**A** left and **B** right) normalised to intracranial volume (ICV) from the HCP-Young Adult dataset. Underweight = BMI < 18.49, normal weight = BMI 18.5 – 24.9, overweight = BMI 25 – 29.9, obese = BMI 30 +. (* = p^corr^ < 0.05, ** = p^corr^ < 0.01).

**Fig. 4:**
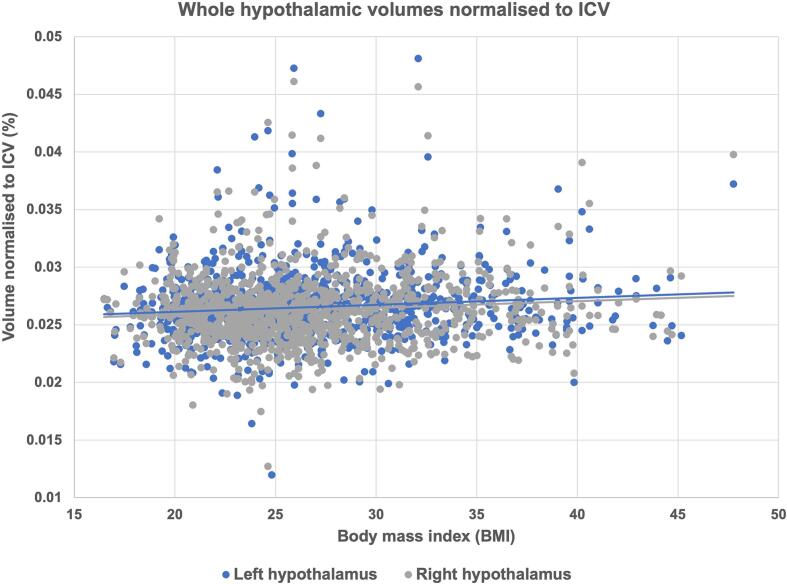
Right and left whole hypothalamic volumes exhibit a significant positive association with BMI (p < 0.05) (*r^2^* = 0.009, 0.012 respectively).

In the HCP-Young Adult dataset (total n = 1111), participants were grouped into underweight = BMI < 18.49 kg/m^2^ (n = 14), normal weight = BMI 18.5 – 24.9 kg/m^2^ (n = 474), overweight = BMI 25 – 29.9 kg/m^2^ (n = 375), obese = BMI 30 + kg/m^2^ (n = 242). For both the obese and overweight groups, whole hypothalamus and multiple hypothalamic nuclei demonstrated significantly larger volume compared to healthy weight participants (Fig. 3). The overweight group exhibited significantly larger left tubular superior nuclei, left whole hypothalami and right tubular inferior nuclei than the control group, with only the left tubular inferior nucleus effect surviving correction for multiple comparisons (p^corr^ < 0.05). When comparing the obese group to controls, almost all individual nuclei and the whole hypothalamic structure bilaterally were significantly larger in those with a BMI over 30 kg/m^2^. After correction for multiple comparisons, the whole right hypothalamus (p^corr^ < 0.01), whole left hypothalamus (p^corr^ < 0.01), left posterior nucleus (p^corr^ < 0.01), left tubular superior nucleus (p^corr^ < 0.05), right posterior inferior nucleus (p^corr^ < 0.05), right posterior nucleus (p^corr^ < 0.05) and right tubular superior nucleus (p^corr^ < 0.05) were significantly larger in the obese group. In all individuals with a BMI over 24.9, the left tubular superior nucleus (p^corr^ < 0.01), whole left hypothalamus (p^corr^ < 0.05), right anterior inferior nucleus (p^corr^ < 0.05), and tubular inferior (p^corr^ < 0.05) and right whole hypothalamus (p^corr^ < 0.05) were significantly greater in volume, with significant effects in the left posterior, left tubular inferior and right tubular superior regions being removed by FDR correction. The underweight group exhibited a smaller right anterior inferior nucleus normalised to ICV than the healthy weight group, but this effect was removed by correction for multiple comparisons. Full statistical results are reported in Table 4. Statistical results for the HCP Young Adult data without correction for ICV or FDR are reported in Supplementary Table 2. The HCP-Young Adult whole hypothalamic data were regressed against BMI and, as may be expected from the between group analyses, a significant relationship (p < 0.05) was identified between greater hypothalamic size and higher BMI values (Fig. 4).

**Table 4:** HCP Young Adults: Hypothalamic nuclei volumetric between group results normalised to ICV.

	Underweight mean (SD)	Normal weight mean (SD)	Overweight mean (SD)	Obese mean (SD)	Underweight – normal weight p-value (FDR)	Overweight – normal p-value (FDR)	Obese – normal p-value (FDR)	BMI > 24.9 – normal weight p-value (FDR)
Hypothalamic volumes (left hemisphere)								
Anterior-inferior	0.0011 (0.0003)	0.0011 (0.0003)	0.0011 (0.0003)	0.0011 (0.0003)	–	–	* (-)	–
Anterior-superior	0.0015 (0.0002)	0.0015 (0.0003)	0.0015 (0.0003)	0.0016 (0.0003)	–	–	* (-)	–
Posterior	0.0069 (0.0007)	0.0073 (0.0009)	0.0074 (0.0010)	0.0076 (0.0011)	–	–	** (**)	* (-)
Tubular inferior	0.0086 (0.0009)	0.0090 (0.0012)	0.0091 (0.0012)	0.0091 (0.0012)	–	–	–	* (-)
Tubular superior	0.0072 (0.0007)	0.0073 (0.0009)	0.0075 (0.0009)	0.0075 (0.0010)	–	** (*)	** (*)	*** (**)
Whole	0.0254 (0.0021)	0.0262 (0.0028)	0.0267 (0.0029)	0.0270 (0.0027)	–	* (-)	*** (**)	** (*)
Hypothalamic volumes (right hemisphere)								
Anterior-inferior	0.0011 (0.0003)	0.0009 (0.0002)	0.0010 (0.0003)	0.0010 (0.0003)	* (-)	–	** (*)	* (*)
Anterior-superior	0.0017 (0.0003)	0.0016 (0.0003)	0.0016 (0.0003)	0.0016 (0.0003)	–	–	* (-)	–
Posterior	0.0073 (0.0008)	0.0076 (0.0011)	0.0075 (0.0012)	0.0078 (0.0012)	–	–	** (*)	–
Tubular inferior	0.0081 (0.0009)	0.0084 (0.0012)	0.0086 (0.0012)	0.0086 (0.0012)	–	* (-)	–	* (*)
Tubular superior	0.0076 (0.0006)	0.0076 (0.0010)	0.0076 (0.0010)	0.0078 (0.0011)	–	–	** (*)	* (-)
Whole	0.0258 (0.0021)	0.0260 (0.0029)	0.0263 (0.0031)	0.0268 (0.0034)	–	–	** (**)	* (*)

Means, standard deviations (SD) and *t*-test p-value results of HCP young adult hypothalamic volumes as normalised^§^ to intracranial volume (ICV) [^§^Hypothalamic volume as a percentage of ICV]; (- = p > 0.05, * = p < 0.05, ** = p < 0.01, *** = p < 0.001). Bracketed significance indicator denotes FDR corrected p-value. Underweight = BMI < 18.49, normal weight = BMI 18.5 – 24.9, overweight = BMI 25 – 29.9, obese = BMI 30 +.

## 4 Discussion

Our initial findings from a modest sample size show that, in obese participants, the anterior-inferior and posterior hypothalamic structures are significantly larger than in matched controls of a healthy weight. Further, we show that in women with AN, reduction in hypothalamus volume compared to controls is proportionate to ICV and most likely a consequence of decreased overall brain size (Walton et al., 2022). In the larger HCP-Young Adult dataset, we replicated the key finding that in overweight and obesity (BMI > 24.9 kg/m^2^), hypothalamus volume is significantly larger. Specifically, the constituent nuclei segmentations reveal that regions containing the arcuate nucleus, PVN and orexin-producing lateral hypothalamus (Billot, 2020) (the posterior, tubular superior and tubular inferior subunits) are the areas responsible for overall greater hypothalamic volume. These regions are known to be the centres of endocrine appetite control (Sohn, 2015).

### 4.1 Imaging the hypothalamus in obesity

As previously highlighted, recently developed automated hypothalamic segmentation tools have facilitated larger scale investigation of the hypothalamus *in vivo* possible. Parcellation of the hypothalamus based on diffusion-weighted MRI data in 100 healthy control participants (27% of which had a BMI of over 30 kg/m^2^) has shown that the anterior–superior hypothalamic subunit, which contains the PVN, exhibits a significant association between mean diffusivity and BMI (Spindler et al., 2020). Increased mean diffusivity is an indicator of higher levels of water in the tissue, which may in turn be representative of oedema or inflammation, although changes in mean diffusivity are a non-specific to this interpretation. These findings may align with a possible explanation of larger hypothalamic and hypothalamic nuclei volume as seen in the present study as being driven in obese individuals by inflammatory processes.

To date, other studies have investigated the segmented hypothalamus *in vivo* in smaller healthy control populations (Baroncini et al., 2012, Makris et al., 2013, Osada et al., 2017, Schindler et al., 2013, Schönknecht et al., 2013), in frontotemporal dementia (Bocchetta et al., 2015, Billot, 2020, Piguet et al., 2011), neurodegenerative disease (Lemaire et al., 2011), schizophrenia (Goldstein et al., 2007), AN (Florent et al., 2020) and major depressive disorder (Wolff et al., 2018). The majority of these studies utilised manual tracing of the hypothalamus, thus limiting sample size. In behavioural variant frontotemporal dementia and Alzheimer’s disease (AD) participants from the ADNI dataset, subunits of the hypothalamus exhibit significant atrophy (Bocchetta et al., 2015, Asan et al., 2021), with one study in behavioural variant frontotemporal dementia showing a relationship between posterior hypothalamic atrophy and disturbed feeding behaviour (Schönknecht et al., 2013). Participants with schizophrenia and non-psychotic first degree relatives exhibited larger hypothalamic volume, particularly at the PVN and mamillary nuclei. In young adults with Prader-Willi syndrome (PWS), a neurodevelopmental condition in part characterised by marked hyperphagia, hypothalamic volumes were significantly smaller than controls and were associated with preoccupation with food (Brown et al., 20222022). While the aforementioned findings in frontotemporal dementia and PWS may seem to portray that smaller hypothalami are linked to overeating, it should be noted that the role of the hypothalamus in the pathology of complex neurodevelopmental disorders or neurodegenerative diseases are likely to be distinct from hypothalamic changes that are due to or a consequence of higher BMI in otherwise healthy individuals. Similarly, many of the studies examining hypothalamic volume in humans focus on diseases or disorders that comprise of an inflammatory component. However, heterogeneity and chronicity of such neuroinflammatory mechanisms, both within and outside of defined pathologies, should be taken into account when aiming to understand bidirectional changes to hypothalamic volume.

The HCP dataset has been used for atlas generation (Neudorfer et al., 2020) and the ADNI dataset was used to validate the contemporary automated Billot *et al.* segmentation tool (Billot, 2020). We present here the first volumetric evidence of hypothalamic changes in obesity, using initially a conservative sample size and subsequently replicating the finding in the larger HCP Young Adult dataset.

### 4.2 An inflammatory perspective of the hypothalamus and obesity

It is known that a high fat diet can induce not just peripheral, but hypothalamic inflammation, which in turn prompts insulin resistance and obesity (Seong et al., 2019). In rodent models, 3 days of a fat rich diet is sufficient to instigate inflammation of the hypothalamus (Thaler et al., 2012) and a correlation has been shown in multiple studies to exist between high dietary fat and hypothalamic inflammatory processes (Milanski et al., 2009, Douglass, 2017, Posey et al., 2009). Thaler *et al.* 2012 reported that hypothalamic inflammation was located primarily at the arcuate nucleus, where the consumption of a high fat diet caused loss of the anorexigenic POMC neurons, thus further unbalancing energy homeostasis and promoting higher satiety thresholds (Thaler et al., 2012). Such a model suggests that hypothalamic inflammation occurs prior to weight gain through a complex signalling cascade. Major aspects of this cascade include the accumulation of activated microglia and astrocytes within the hypothalamus (Jais and Brüning, 2017201720172017), and increased expression of pro-inflammatory cytokines, such as interleukin 1β, interleukin 6 and tumour necrosis factor (TNF)-α, in the hypothalamus (De Souza et al., 2005). The presently reported larger hypothalamic volume in obese participants align with the notion of hypothalamic inflammation and gliosis (i.e. astrocyte hypertrophy and microglial proliferation). Nevertheless, as cellular or inflammatory processes cannot be inferred from morphometric data, future research with complementary methods (e.g., magnetic resonance spectroscopy, gene expression) will be useful in delineating the potential drivers of altered hypothalamic volume.

Another candidate member of the inflammatory cascade in obesity-related hypothalamic inflammation is the blood–brain barrier (BBB). The arcuate nucleus is adjacent to the median eminence, a highly vascularised structure with a permeable BBB, making it sensitive to circulating nutritional information and pro-inflammatory cytokines (Williams, 2012). BBB alterations have been reported in rodents in response to high dietary fat and in patients with type II diabetes (Yi et al., 2012), and the expression of BBB tight junction proteins is reduced in response to high calorific food intake, in turn prompting further microgliosis (Freeman and Granholm, 2012). The overall disruption of the BBB allows for leukocyte infiltration to the brain (Varvel et al., 2012), exacerbating the inflammatory response. The arcuate nucleus’ sensitive and permeable BBB makes it especially vulnerable to inflammation, which may be reflected in the present findings of greater volume particularly of this hypothalamic sub-structure.

Evidence of localised neuroinflammation in the hypothalamus is not limited to the arcuate nucleus. Pro-inflammatory cytokines induced by high fat diet access the brain at the mediobasal hypothalamic site, initiating activation of cytokine receptors (Cai and Liu, 2012), and pro-inflammatory cascades promote central inflammation that stimulate projecting neurons (Blatteis, 2007). Our findings of a moderate reduction in size of the hypothalamus in AN compared to matched controls, an effect which was removed after correction, supports the hypothesis that specifically a high fat diet induces inflammation and therefore may result in larger volume of the hypothalamus. However, these results do not exclude the possibility of a hypothalamic volume-based predisposition to altered food intake in AN or obesity.

### 4.3 The hypothalamus and anorexia nervosa (AN)

In AN, it has been theorised that the hypothalamus plays a key role, particularly given the numerous clinical features indicative of aberrant hypothalamic-pituitary functioning, such as hypercortisolaemia and amenorrhea. Preclinical models of activity-based anorexia have further shown disturbances in neuropeptide function, hypothalamic degeneration and inflammation. In humans, examination of hypothalamic resting state functional connectivity using manual landmark-based segmentation (Matsuda et al., 1999) showed that participants with AN had reduced connectivity between the hypothalamus and putative reward regions relative to healthy controls in response to water infusion (Simon et al., 2020). This extends findings of perturbed arcuate nucleus and lateral hypothalamic structural connectivity in AN (Florent et al., 2020), which could reflect aberrant glutamatergic response to food intake. Hypothalamic inflammation is known to be a driving force in the development of anorexia-cachexia syndrome (van Norren et al., 2017), and thereby may be hypothesised in AN to be linked to larger hypothalamic volume. However, similar to the results presented in the present study, despite reported differences in hypothalamic metabolic function and both resting-state and structural connectivity in AN, Simon *et al.* identified no significant differences in hypothalamic volume (normalised to total brain volume) between the AN group and the healthy-weight controls. The ENIGMA Consortium’s AN Working Group highlighted in a large *meta*-analysis that individuals with AN have sizable reductions in cortical thickness and subcortical volumes compared to healthy control participants (Walton et al., 2022). As these findings also did not normalise regional volume for ICV, they might suggest that chronic malnutrition relates to global reductions in brain size. Indeed, our findings of a significant decrease in size of the hypothalamus which is not present after normalisation to ICV indicates that hypothalamic volume alteration in AN is proportional to more generalised brain volume loss.

## 5 Limitations

We present findings of altered hypothalamic structure in overweight and obese populations, and to a lesser extent, in participants who are underweight. However, without longitudinal study, it is not known whether larger hypothalamic volume is a cause or effect of obesity, especially given the known complex role of the structure in appetite and satiety. We theorize that greater volume seen in this human neuroimaging investigation may be due to hypothalamic inflammatory processes, in particular gliosis and astrocyte hypertrophy, but further research is needed to establish the relationship between hypothalamic volume and inflammatory markers. Additional microstructural and spectroscopic investigation of the hypothalamus in obesity may also support the existence of regional inflammatory processes. Furthermore, we find that segmented substructures of the hypothalamus containing the PVN, arcuate nucleus and orexin-producing lateral regions are particularly affected volumetrically in participants with higher BMI, however it should be noted that delineations of the automated algorithm do not segment these regions in an isolated manner. Therefore, attaining further granular detail of the hypothalamic nuclei in the future may allow for clearer conclusions with regards to volumetric alterations due to neuronal population changes. Potential differences in hypothalamic volume between males and females were not a focus of this investigation, however, it should be considered that sexual dimorphism may affect hypothalamic volume in young adults and may differentially affect the hypothalamic volumetric relationship to weight and food intake. Given the known existence of complex and multifactorial sexual disparity in the prevalence of AN, BN (Striegel-Moore et al., 2009) and obesity (Cooper et al., 2021), this is an important avenue for further research.

## 6 Conclusions

We report that in groups with high BMI, whole hypothalami and segmented regions containing the satiety and food regulatory constituent nuclei are significantly larger in volume. Moreover, an associative relationship exists between BMI and hypothalamic volume, with greater volume scaling with higher BMI. Given the extensive evidence of a high fat diet-induced mechanism of hypothalamic inflammation in obesity, we speculate that larger size of the hypothalamus, particularly the PVN and arcuate nuclei containing regions which control food intake, may be due to localised neuroinflammation in people who are obese. However, the design of this study does not extend to the inference of a causal relationship, and we suggest that further research is needed to establish whether larger hypothalamic volume is a cause or effect of higher than normal BMI. The inflammatory cascade of increased cytokine expression, diet-induced gliosis, BBB disturbance and vascular alterations may exacerbate further dysregulation of energy homeostatic mechanisms governed by the hypothalamus, thus further challenging weight-loss strategies. We posit that future research should endeavour to clarify and identify the causes of greater hypothalamic volume in obesity, for the purposes of addressing therapeutic opportunities for a world-wide public health need.

## CRediT authorship contribution statement

**Stephanie S.G. Brown:** Formal analysis, Investigation, Methodology, Conceptualization. **Margaret L. Westwater:** Data curation, Project administration. **Jakob Seidlitz:** Data curation, Project administration. **Hisham Ziauddeen:** Data curation, Project administration. **Paul C. Fletcher:** Funding acquisition, Project administration, Investigation, Conceptualization, Supervision.

## Declaration of Competing Interest

The authors declare that they have no known competing financial interests or personal relationships that could have appeared to influence the work reported in this paper.

## Data Availability

Data will be made available on request.
