# Supplementary material for: Hypothalamic volume is associated with body mass index
Source: Neuroimage Clin. 2023 Jul 24;39:103478. doi: 10.1016/j.nicl.2023.103478 (PMC10509524; doi:10.1016/j.nicl.2023.103478)
Supplement: Supplementary data 1 [file mmc1.docx]

|  | SUPPLEMENTARY Table 1: Hypothalamic nuclei volumetric between group results without normalisation to ICV | | | | | | | | | |  |
| --- | --- | --- | --- | --- | --- | --- | --- | --- | --- | --- | --- |
|  | | **Control mean (SD)** | **Overweight mean (SD)** | **Obese mean (SD)** | **BN mean (SD)** | **AN mean (SD)** | **AN/BN matched control mean** | **Overweight-Control p-value** | **Obese-Control p-value** | **BN-Control p-value** | **AN-Control p-value** |
| Hypothalamic nuclei volumes | |  |  |  |  |  |  |  |  |  |  |
| Left anterior-inferior | | 18.45 (3.80) | 19.13 (2.97) | 20.25 (3.33) | 16.72 (4.65) | 16.61 (4.29) | 17.67 (5.20) | - | ** | - | - |
| Left anterior-superior | | 25.97 (4.05) | 25.97 (3.84) | 25.57 (4.70) | 22.04 (3.36) | 21.13 (4.77) | 23.01 (3.85) | - | - | - | - |
| Left posterior | | 120.90 (16.39) | 120.68 (15.03) | 121.39 (15.18) | 120.37 (13.78) | 117.23 (10.23) | 122.38 (15.60) | - | - | - | - |
| Left tubular inferior | | 136.90 (19.73) | 140.32 (13.25) | 143.71 (19.86) | 146.45 (13.49) | 145.86 (13.41) | 145.73 (14.36) | - | - | - | - |
| Left tubular superior | | 115.05 (12.90) | 117.38 (13.25) | 113.30 (18.31) | 113.76 (14.04) | 108.83 (9.72) | 117.97 (12.84) | - | - | - | ** |
| Left whole | | 417.28 (42.15) | 423.47 (35.21) | 424.22 (46.20) | 419.33 (25.46) | 409.66 (25.62) | 426.76 (38.82) | - | * | - | - |
| Right anterior-inferior | | 26.52 (3.67) | 16.53 (3.19) | 16.75 (3.23) | 14.50 (3.74) | 14.92 (3.08) | 14.26 (3.39) | - | - | - | - |
| Right anterior-superior | | 128.59 (14.12) | 26.48 (4.74) | 25.07 (4.89) | 23.61 (4.34) | 21.95 (3.80) | 24.60 (4.23) | - | - | - | * |
| Right posterior | | 114.25 (15.52) | 118.51 (14.93) | 124.04 (15.71) | 119.73 (16.16) | 118.57 (9.70) | 123.99 (15.02) | * | ** | - | - |
| Right tubular inferior | | 128.58 (14.12) | 132.99 (15.62) | 130.69 (18.73) | 129.41 (10.87) | 127.89 (11.70) | 132.78 (12.63) | - | - | - | - |
| Right tubular superior | | 124.09 (14.06) | 126.78 (17.30) | 121.42 (19.33) | 118.52 (15.37) | 113.83 (13.61) | 119.95 (9.93) | - | - | - | - |
| Right whole | | 409.38 (37.71) | 421.31 (44.93) | 417.97 (45.86) | 405.80 (31.52) | 397.15 (29.26) | 415.58 (28.28) | * | - | - | * |

**SUPPLEMENTARY Table 1:** Means, standard deviations (SD) and t-test p-value results of raw segmented hypothalamic volumes uncorrected for multiple comparisons (- = p > 0.05, * = p < 0.05, ** = p < 0.01, *** = p < 0.001)

| SUPPLEMENTARY Table 2: HCP young adult hypothalamic nuclei volumetric between group results without normalisation to ICV | | | | | | | | |
| --- | --- | --- | --- | --- | --- | --- | --- | --- |
|  | **Underweight mean (SD)** | **Normal weight mean (SD)** | **Overweight mean (SD)** | **Obese mean (SD)** | **Underweight – normal weight p-value** | **Overweight – normal p-value** | **Obese – normal p-value** | **BMI > 24.9 – normal weight p-value** |
| Hypothalamic nuclei volumes |  |  |  |  |  |  |  |  |
| Left anterior-inferior | 16.66 (5.39) | 16.28 (4.32) | 17.10 (4.80) | 17.12 (4.57) | - | ** | * | ** |
| Left anterior-superior | 23.02 (3.02) | 23.83 (4.46) | 24.69 (4.46) | 24.57 (4.56) | - | ** | * | ** |
| Left posterior | 103.39 (13.44) | 114.04 (15.62) | 118.27 (16.05) | 118.09 (15.84) | - | *** | ** | *** |
| Left tubular inferior | 128.40 (16.15) | 139.29 (18.85) | 146.03 (18.15) | 142.71 (19.19) | * | *** | * | *** |
| Left tubular superior | 108.49 (12.22) | 113.62 (15.98) | 120.34 (15.55) | 117.50 (17.02) | - | *** | ** | *** |
| Left whole | 380.16 (40.81) | 407.06 (45.14) | 426.42 (44.35) | 419.99 (45.40) | * | *** | *** | *** |
| Right anterior-inferior | 15.93 (5.02) | 14.84 (4.30) | 15.78 (3.93) | 15.84 (4.77) | - | ** | ** | *** |
| Right anterior-superior | 25.35 (5.02) | 24.00 (4.75) | 24.98 (4.73) | 24.87 (4.90) | - | ** | * | ** |
| Right posterior | 109.32 (13.80) | 117.02 (15.91) | 119.71 (16.87) | 120.84 (16.15) | * | * | ** | ** |
| Right tubular inferior | 121.68 (16.32) | 130.29 (19.57) | 137.27 (18.97) | 133.47 (19.50) | - | *** | * | *** |
| Right tubular superior | 114.04 (8.90) | 117.15 (14.74) | 121.89 (15.12) | 120.91 (16.10) | - | *** | ** | *** |
| Right whole | 386.32 (36.95) | 403.31 (43.00) | 419.63 (43.32) | 415.94 (45.44) | - | *** | *** | *** |

**SUPPLEMENTARY Table 2:** Means, standard deviations (SD) and t-test p-value results of raw segmented hypothalamic volumes uncorrected for multiple comparisons (- = p > 0.05, * = p < 0.05, ** = p < 0.01, *** = p < 0.001).
